# Blood collection under anesthesia, peripheral blood cells, plasma biochemistry, and plasma protein electrophoresis in a living fossil: the Spotted Ratfish (*Hydrolagus colliei*)

**DOI:** 10.3389/fvets.2023.1305968

**Published:** 2024-01-11

**Authors:** Pablo Morón-Elorza, Hugo David, Hugo Batista, Vanessa Quina, Nuria Baylina, Nuno Pereira

**Affiliations:** ^1^Department of Pharmacology and Toxicology, Faculty of Veterinary Medicine, Universidad Complutense de Madrid, Madrid, Spain; ^2^Research Department, Fundación Oceanogràfic, Valencia, Spain; ^3^Biology and Conservation, Oceanario de Lisboa, Lisboa, Portugal

**Keywords:** *Chimaeran*, chondrichthyan, fish, hematology, blood chemistry, chemical immobilization, electrophoretogram

## Abstract

This report describes the safe and effective handling under anesthesia, blood collection and main clinical pathology values determination for three adult Spotted Ratfish (*Hydrolagus colliei*) (two males and one female) successfully maintained under human care for more than 20 years. The anesthetic MS-222 diluted in tamponed salt water at 50 ppm provided deep narcosis with mean induction and recovery times of 5 and 20 min, allowing safe handling and blood collection in the three animals sampled. Major leukocyte types were similar to other teleost and elasmobranch species, identifying lymphocytes as the predominant leukocyte (75.5%), followed by the fine eosinophilic granulocytes (10.25%), the coarse eosinophilic granulocytes (9.75%), and a small percentage of monocytes (5.50%). Plasma biochemistry values in this species were like those seen in elasmobranchs, with the highest levels of blood urea nitrogen described in any *Chondrichthyes* (mean 679.7 mmol/l). Plasma protein electrophoresis analysis in the Spotted Ratfish showed five consistent fractions, like those previously described in other fish species, with a negligible quantity of proteins migrating in the region equivalent to albumin, and with fraction 4 (mean 53.20%) as the predominant fraction. Despite the limitations imposed by the small sample size and the challenging access to the studied species under human care, this study will shed light on and enhance clinical knowledge regarding Ratfish handling, anesthesia, blood collection, and analysis. It aims to deliver a comprehensive clinical pathology description, presenting valuable data for professionals engaged in the care and management of *Chimaerans*.

## Introduction

Living holocephalans (Subclass: *Holocephali*) are commonly referred to as ghost fish, chimaeroid fish, or ratfish, and are the closest relatives to sharks and rays. They are an ancient lineage that evolved over 300 million years ago and the physiology and morphology of living representatives differs little from their fossil ancestors (1, 2). While public aquariums and marine research facilities frequently keep sharks and rays as emblematic species, increasing information about their husbandry and medicine, members of the order *Chimaeriformes* are much less frequently kept in aquariums (2).

Currently, there are still few published studies on chondrichthyan hematology and biochemistry, with only few values published in sharks and rays, showing large interspecific differences (1, 3–5). Furthermore, clinical pathology studies involving Holocephalans have only provided brief preliminary results, mainly using small numbers of wild-caught individuals, which were sampled directly after capture and survived only for short periods (6, 7).

The fact that most Chimeroid species occur in deep waters makes them less suitable for aquariums and *ex-situ* research facilities (2). The Spotted Ratfish (*Hydrolagus colliei*) is a notable exception, as it occurs in shallow waters of the western coast of Canada and the USA (1). This, together with the recent increase in the knowledge of their biology, husbandry, and their management is slowly increasing the number of Spotted Ratfish held in public aquariums (2). Medical results obtained from chimeras successfully maintained in aquariums can help fill the gap in the basic knowledge of hematology, blood chemistry, and plasma protein electrophoresis (PPE) in this still enigmatic group of animals.

## Materials and methods

### Animals and environmental conditions

In the context of a health management program, blood was collected from 3 adult Spotted Ratfish (*Hydrolagus colliei*) (one female and two males) maintained under human care at Oceanário de Lisboa, a Public Aquarium in Portugal (www.oceanario.pt) (Supplementary Figure 1). Such a small number of individuals were included in the study as the Spotted Ratfish is still a rare species to be maintained under human care, and access to the species is very limited. Their difficult acquisition and challenging husbandry make them much less studied in the scientific literature, turning the veterinary examination of these three individuals into a great opportunity to provide basic knowledge on their handling and complementary diagnosis, increasing the bloodwork database in Holocephalans (2). Clinical physical examination was normal, and no signs of disease were detected in any of the animals included in the study. Age and morphological details for the three animals are provided as Supplementary Table 1. All three animals sampled were considered adults according to previous studies determining maturity size in the Spotted ratfish 202.8 mm for females and 157.2 mm for males) (1).

All ratfish were maintained in a trapezoidal 15, 800 L tank with a depth of 1.9 meters, in semi-darkness (the tank was illuminated with 2 fluorescent lights (Osram T8 36W coral plus and Osram T8 36W natura) with 13 h of light daily. All Spotted Ratfish have been kept in the same tank of the aquarium for over 18 years. They were fed 80 grams of shrimp, 40 grams of squid, 40 grams of mussel, 40 grams of sprat and 40 grams of smooth clam weekly, divided in three feedings throughout the week. This sums a total of 273.04 kcal, 37.13 g of protein and 7.32 g of fat per week. The tank contained artificially generated salt water, and environmental parameters were: 9.5°C, 32 g/L salinity and 8.1 pH. The system has a mechanical, chemical, and biological filtration system with UV disinfection, one protein skimmer and a heat exchanger for temperature control. The waterflow through the filtration system was 9–11 m^3^/h Ammonium was kept at 0.001 ppm, nitrite was kept under 0.004 ppm and nitrate under 4.6 ppm. Ratfish were fed 3 days per week with 80 g per feeding of a mixture of thawed squid, shrimp, herring, sprat, capelin, and clam. Diet was supplemented with vitamin C, 20 mg/kg, once a week and elasmobranch multivitamin and mineral in-house formulated and prepared by Premix (4935-232 Viana do Castelo, Portugal) twice a week.

### Sampling procedure

For sampling, the three animals were handled individually on three separate interventions. The environment was illuminated with low red light to minimize the stress associated with photosensitivity (2). All handling was conducted in a 200 L tank under deep narcosis using tricaine methane sulfonate (MS-222) (Tricaine PHARMAQ^®^) at 50 ppm, which was buffered using sodium bicarbonate at a 1:2 ratio (8, 9).

The blood collection site was selected after examining a dead specimen of a similar species, *Chimera monstrosa* (Supplementary Figure 2). A volume of 0.7 mL−1 mL peripheral blood was collected from the caudal blood vessel via venipuncture of the caudal hemal arch (Supplementary Figure 3) using a previously heparinized 23-gauge needle attached to a 1 ml syringe, in a lateral approach, dorsocaudal to the pelvic fins. Once blood was collected, it was directly transferred to a 1 ml tube containing lithium-heparin as an anticoagulant.

After blood sampling, the following morphometrical measures were collected using a dynamometer and a measuring tape: total length (TL, snout to tip of the tail), snout-vent (SV, snout to vent) and weight.

### Sample analysis

Packed cell volume (PCV) was determined using microhematocrit capillary tubes, which were centrifuged at 15, 870 g for 5 min using a Centurion Pro-Vet HE centrifuge (Centurion Scientific Ltd., PO18 9JL Chichester, UK). Hemoglobin was measured using a cyanide-free colorimetric method (Mindray^®^ BC-5000 Auto Hematology Analyzer; Uranolab; Sintra 2710-297, Portugal). Plasma total solids were measured only in the third animal due to equipment availability, using a manual refractometer (LABOLAN^®^ model FG301/311; Labolan S.L., Esparza 31191, Spain).

Erythrocytes, total leukocytes, and thrombocytes were counted in an improved Neubauer chamber (Assistent GLASWARENFABRIK KARL HECHT GMBH and CO KG; 97647 Sondheim vor der Rhön, Germany) using an optical microscope (DM2000, Leica) at 40 x magnification. A 1:100 solution was prepared, diluting 20 μl of blood in 1980 μl Rees Ecker Fluid (Atom Scientific Ltd., Hyde SK14 4GX, UK). Counting was repeated by diluting a 10 μl blood sample in 490 μl Natt–Herricks solution (1:50 dilution) (Natt-Pette^TM^, Exotic Animal Solutions, Inc., 32941 Melbourne, USA). These dilutions were performed following the guidelines described for hematology analysis in exotic animal species, specifically for Chondrichthyans, and allowed us to achieve adjacent, non-overlapping cells in the standard grid area of the Neubauer chamber (10, 11). This study presents results from both diluents, intending to facilitate additional comparisons. Blood smears were performed using the blood stored in the heparin (< 5 min since blood collection), left to dry at room temperature (22°C). Striving to contribute to the standardization Leica of hematological analysis for peripheral leukocytes in *Chondrichthyes*, Spotted Ratfish leukocytes were differentiated based on morphology using a Romanowsky stain variant (Diff-Quick, Maim S.L., 08500 Barcelona, Spain)and classified as lymphocytes, monocytes, fine eosinophilic granulocytes (FEG), which due to their granule morphology and staining properties resemble avian heterophils, and coarse eosinophilic granulocytes (CEG), which resemble avian eosinophils (10, 12). Leukocyte differentials were determined by counting a minimum of 200 WBC per animal and sample, and the further calculation of the cell percentages. Total cell counts for the leukocyte differential counts were obtained applying the percentages to the total WBC count. Images were obtained using a digital camera (MC170 HD, Leica, Wetzlar, Germany) connected to an optical microscope (DM2000) and cell measures were determined using commercial software (Leica Application Suite, version 4.6.0, Leica).

A volume of 0.5 ml blood from the lithium-heparin tube was introduced into an IDEXX Catalyst OneTM chemistry analyzer (IDEXX Laboratories, Inc.), in which plasma was isolated via centrifugation (11, 600 x cg for 90 s) and glucose was directly measured (total time since blood collection was always under 10 min). The spare plasma was sent to DNAtech Laboratory (Estrada do Paço do Lumiar 22, building E, first floor, 1649-038 Lisboa) for the determination of the rest of the plasma chemistry analytical values (using a Beckman Coulter DxC 700 AU; Beckam Coulter Inc., 2794-044 Carnaxide, Portugal) and included alkaline phosphatase (ALP), aspartate aminotransferase (AST), blood urea nitrogen (BUN), calcium (Ca), total cholesterol, chloride (Cl), creatine phosphokinase (CPK), lactate dehydrogenase (LDH), sodium (Na), phosphorus, potassium (K), and triglycerides (Trig). Plasma protein electrophoresis was also performed (using a Sebia Hydrasys; Sebia Portugal, 2740-244 Porto Salvo, Portugal). The Spotted Ratfish PPE fractions were classified by numerical designation following the criteria established in previous studies with teleost and elasmobranch fish (13).

## Results

As previously described in wild-caught Spotted Ratfish, weight was higher in the female (1, 350 g) compared with the two males sampled (650 and 560 g) (1). The descriptive morphometric data for the three animals sampled is presented as Supplementary Table 1.

Anesthesia induction produced a short initial excitement phase that quickly evolved to a deep sedation plane. A longer period was necessary to reach deep narcosis and recovery. The entire procedure, from introduction in the anesthetic bath to recovery in a side tank lasted between 13 min and 28 min depending on the animal. Detailed anesthetic times have been included as Supplementary Table 2.

**Figure 1:**
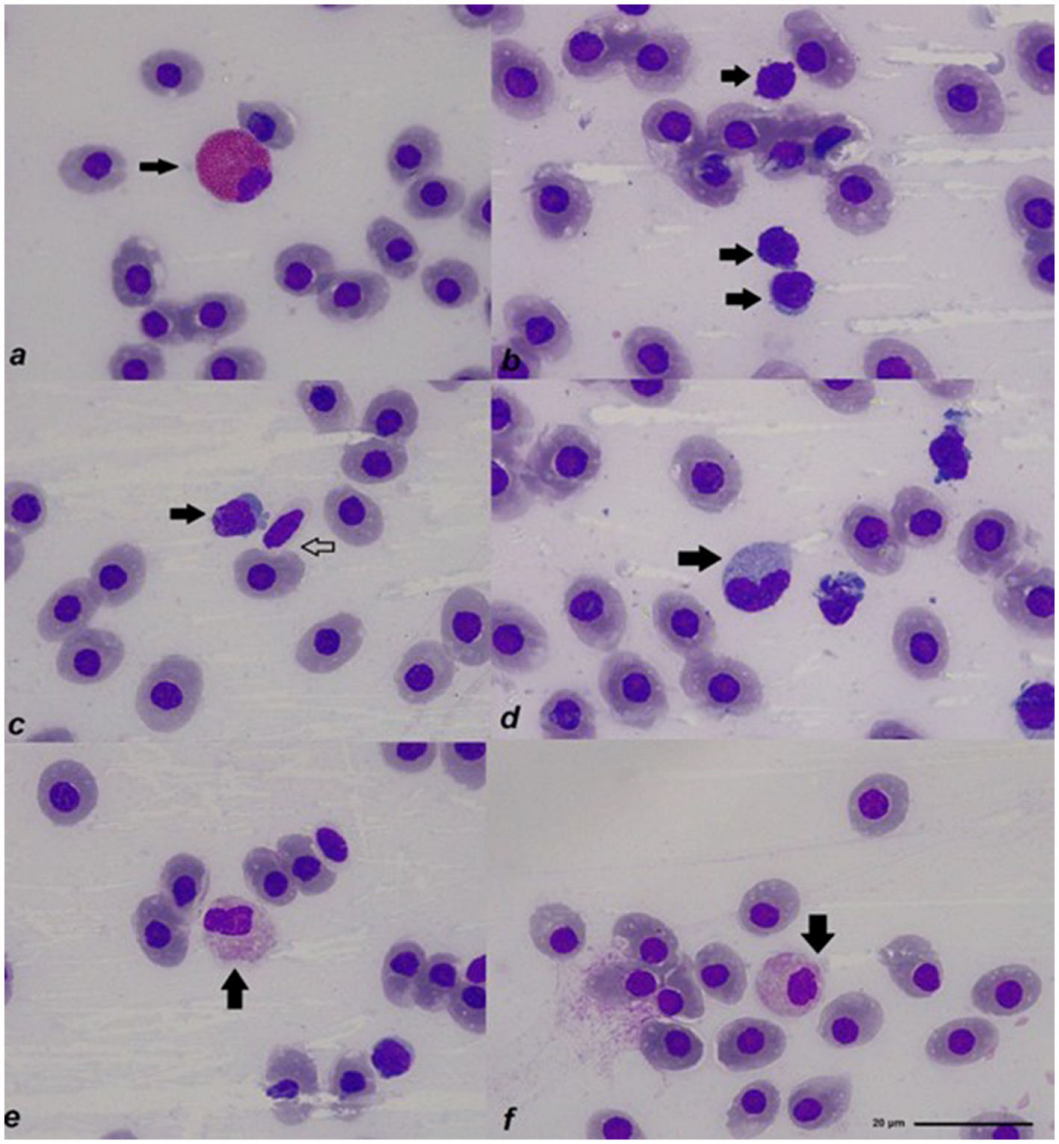
Main peripheral blood cells of the Spotted Ratfish (*Hydrolagus colliei*): **(a)** Coarse eosinophilic granulocyte (arrow); **(b)** lymphocytes (arrows); **(c)** lymphocyte (solid arrow) and thrombocyte (hollow arrow); **(d)** monocyte (arrow); **(e)** fine eosinophilic granulocyte (arrow); **(f)** fine eosinophilic granulocyte (arrow); all photos were taken from blood smears stained with Diff-Quick, in a 1000x magnification.

In the Spotted Ratfish, Rees Ecker solution made lymphocytes visible for the total WBC count and allowed the counting of thrombocytes. Natt–Herricks solution made granulocytes brighter and easier to spot between all the cells. Main peripheral cell morphology resulted similar to what has been reported in most elasmobranchs and is described as Supplementary Table 3. Examples of Spotted Ratfish peripheral blood cells can be seen in Figure 1. A detailed list of results for the main hematology and plasma chemistry parameters obtained in the three studied animals can be seen in Tables 1, 2.

**Table 1:** Hematology values for the three adult Spotted Ratfish (*Hydrolagus colliei*).

**Hematology parameter**	**Animal 1**	**Animal 2**	**Animal 3**	**Mean**	**Median**
PCV (%)	15	14	17	15.33	15
TS (g/dl)	-	-	6.4	NA	NA
RBC count manual (Rees-ecker) (10^12^cells/l)	0.60	0.74	0.67	0.67	0.67
RBC count manual (Natt–Herricks) (10^12^cells/l)	0.53	0.68	1.00	0.62	0.66
WBC count manual (Rees-ecker) (10^9^cells/l)	22.25	27.50	13.75	21.17	22.25
WBC count manual (Natt–Herricks) (10^9^cells/l)	25.12	41.00	21.75	29.29	25.12
WBC count manual (Average) (10^9^cells/l)	23.68	34.25	17.75	25.23	23.69
L (%)	68.25	75.50	79.50	74.42	75.50
M (%)	4.50	5.50	7.50	5.83	5.50
N (%)	0	0	0	0	0
FEG (%)	11.75	10.25	10.25	10.75	10.25
CEG (%)	15.50	9.75	2.75	9.33	9.75
B (%)	0	0	0	0	0
L count (10^9^cells/l)	16.16	25.86	14.11	18.71	16.16
M count (10^9^cells/l)	1.06	1.88	1.33	1.42	1.33
N (10^9^cells/l)	0.00	0.00	0.00	0.00	0.00
FEG (10^9^cells/l)	2.78	3.51	1.81	2.70	2.78
CEG (10^9^cells/l)	3.67	3.3	0.48	2.49	3.33
B (10^9^cells/l)	0.00	0.00	0.00	0.00	0.00

PCV, packed cell volume; TS, total solids in plasma; NA, not applicable; RBC, red blood cell; WBC, white blood cell; L, lymphocyte; M, monocyte; N, neutrophil; FEG, fine eosinophilic granulocyte; CEG, coarse eosinophilic granulocyte.

**Table 2:** Plasma chemistry values for the three adult Spotted Ratfish (*Hydrolagus colliei*).

**Biochemistry parameter**	**Unit**	**Animal 1**	**Animal 2**	**Animal 3**	**Mean**	**Median**
ALP	U/l	57.4	45.9	33.5	45.6	45.9
AST	U/l	38.0	39.0	29.0	35.3	38.0
BUN	mg/dl	2, 163.2	1, 604.5	1, 903.5	1, 890.4	1, 903.5
	mmol/l	772.5	573.0	679.7	679.7	679.7
Calcium	mg/dl	15.0	16.3	14.3	15.2	15.0
	mmol/l	3.74	4.07	3.57	3.79	3.74
Cholesterol	mg/dl	229.0	253.0	243.0	241.6	243.0
	mmol/l	5.9	6.5	6.3	6.2	6.3
CPK	(U/l)	2, 737.0	3, 341.0	738.0	2, 272.0	2, 737.0
Chloride	mmol/l	291.0	304.0	276.0	290.3	291.0
Glucose	mg/dl	128.0	85.0	100.0	104.3	100.0
	mmol/l	7.1	4.7	5.5	5.8	5.5
Potassium	mmol/l	4.18	4.57	4.41	4.39	4.41
LDH	U/l	32.0	57.0	85.0	58.0	57.0
Sodium	mmol/l	287.0	299.0	289.0	289.7	289.0
Phosphorus	mg/dl	5.4	6.1	5.1	5.6	5.4
	mmol/l	1.74	1.97	1.64	1.78	1.74
Triglycerides	mg/dl	479.0	488.0	362.0	443.0	479.0
	mmol/l	5.46	5.56	4.13	5.05	5.46

ALP, alkaline phosphatase; AST, aspartate aminotransferase; BUN, blood urea nitrogen; CPK, creatinine phosphokinase; LDH, lactate dehydrogenase.

Spotted Ratfish electrophoretograms were similar to those previously reported in other elasmobranch and teleost species and could be divided into five well-defined fractions (13–16). Plasma protein electrophoresis analysis could not be performed in the third animal due to insufficient plasma. Results for the first two animals are shown in Table 3 and as Supplementary Figure 4.

**Table 3:** Plasma protein electrophoresis values for two Spotted Ratfish (*Hydrolagus colliei*).

**PPE parameter**	**Animal 1**	**Animal 2**	**Mean**
Total proteins (g/dl)	2.70	3.20	2.95
Fraction 1 (%)	4.40	4.40	4.40
Fraction 2 (%)	5.70	5.00	3.15
Fraction 3 (%)	16.80	16.00	16.40
Fraction 4 (%)	66.00	64.40	53.20
Fraction 5 (%)	7.10	10.00	8.55
Fraction 1 (g/dl)	0.10	0.10	0.10
Fraction 2 (g/dl)	0.20	0.20	0.20
Fraction 3 (g/dl)	1.30	0.50	0.90
Fraction 4 (g/dl)	1.80	2.10	1.55
Fraction 5 (g/dl)	0.20	0.30	0.25
Albumin/Globulin ratio	0.05	0.05	0.25
Fraction 3:4 ratio	0.25	0.25	0.25

Please note that for the determination of albumin/globulin ratio, fraction 1 was considered equivalent to albumin while fractions 2–4 were considered equivalents to globulins.

## Discussion

This technical brief research report intends to provide information on MS-222 anesthesia and the main clinical pathology values in the Spotted Ratfish. The primary limitation of this study was the unfeasibility of statistical analysis due to the small sample size, a challenge inherent in working with Chondrichthyans, and notably more pronounced with Chimeras. Nevertheless, the privilege of having access to samples from this uncommon species under controlled conditions enabled the rare opportunity to establish clinical values. These findings will be of great use for subsequent comparative studies and may serve as an initial reference until more expansive research with homogenous populations can be conducted. We hope that the publishing of this study will encourage other colleagues to replicate it and publish their results allowing the buildup of a database of clinical pathology in *Chimaerans*.

Contrary to what is reported in the literature for fish, we recorded a first initial period of excitement before reaching the light sedation plane (17). The dosage of 50 ppm of MS-222, neutrally buffered using sodium bicarbonate (1:2), produced a safe plane of anesthesia that allowed for stressless handling of the animals and precise execution of the blood collection. Other reports of anesthesia with MS-222 in this species used concentrations ranging from 50 ppm to 133 ppm but none describe times for induction or recovery (18). As it has been described in other anesthesia studies involving teleost fish, our data suggests that longer induction times required until the onset of narcosis resulted in a longer recovery time (19).

Hematology analysis techniques adapted for elasmobranchs produced good results in this species, being the Spotted Ratfish cell morphology similar to that previously described in sharks and rays (10). Both Rees Ecker and Natt–Herricks served to perform the complete hematological counts, and further studies with larger populations should be developed to statistically evaluate the differences obtained in the results; because of this, this brief report provides values obtained using both solutions. A limitation to this study that should be considered was that neither the Natt–Herricks nor the Rees Ecker dilutants were modified to increase osmolality to resemble that of holocephalans body fluids, which has proven to be similar to that of elasmobranchs, being slightly hyperosmotic to saltwater (7). An adjustment in the osmolality of Natt–Herricks stock solution could have been performed to reduce the risk of cell lysis (10, 20).

In our study, RBC and WBC counts were directly performed (< 30 min) after blood collection and dilution using both cell counting formulas, minimizing cell lysis, which was not appreciated in any of the ratfish samples during counting. Moreover, we were unable to locate any references describing the effects of Rees Ecker on elasmobranch blood. At our institution, we have consistently utilized it for over 6 years in a wide range of elasmobranch species, leading to intact cells and hematological results that align with the ranges described in publications utilizing the Natt–Herricks modified solution (David, H *pers com*). Nevertheless, readers should note that the lack of precision in manually determining RBC and WBC counts has been proven in elasmobranch and teleost species. While these manual counts can offer a rough estimate of red blood cell numbers and their publication may be crucial for future comparisons, they fall short in providing the level of accuracy required for assessing anemia or calculating precise mean corpuscular volume (MCV) and mean corpuscular hemoglobin (MCH) values (11).

The Spotted Ratfish presented lower PCV values (15%) than those reported in most teleost and Chondrichthyans; total RBC were higher than those published in other *Chondrichthyes*, though were kept between values reported in teleost species and were similar to the PCV reported for the holocephalan *Chimera monstrosa* (15.7%) (6, 10). Total WBC counts obtained in the Spotted Ratfish (22.25 × 103 cells/μl) were within the previously published values in the different species of Chondrichthyes studied, which show great interspecific variations and range from 4.14 × 103 to 50.7 × 103 cells/μl in apparently healthy animals (10). Total solids in plasma, peripheral leukocyte percentages, and total cell count for each leukocyte showed values similar to those found in other *Chondrichthyes* (10, 15, 21).

Clinical pathology investigations on Holocephalans up to date have yielded only concise initial findings, primarily employing limited numbers of specimens captured from the wild (6, 7). As it has been well documented in elasmobranchs, capture trauma and capture-related stress can lead to significant alterations in analytical data, including hematology and plasma chemistry (22, 23). These alterations underscore the importance of meticulously detailing capture procedures and sampling techniques when establishing blood values in elasmobranchs. This is crucial to prevent the influence of stress related to capture on analytical data (11, 23). To the authors' knowledge, this is the first study determining blood analytics for any Holocephalan species maintained under human care, which allowed for fast and efficient capture and sampling, and may aid in future health assessment of other *Chimaerans* held under human care.

Median values provided for electrolytes as well as ALP, AST, and LDH resulted very similar to those previously reported in many shark and ray species (5, 15, 21, 24). Creatine phosphokinase levels determined in this study were greater than those values previously determined in other *Chondrichthyes*. This intracellular enzyme, which seems to rapidly increase in elasmobranchs associated with stress, handling and short-burst swimming, has shown significant interspecific and interindividual variations with greater values described in deep-water elasmobranchs (25).

Median cholesterol (6.3 mmol/l), BUN (679.7 mmol/l), and triglyceride (5.46 mmol/l) levels were greatly over those reported in previous studies with elasmobranchs for these analytes (4, 5, 15, 21, 24). Variations related to sex, season, reproductive stage, and food intake have been described in these analytes in teleost and elasmobranch fish (26, 27). Despite body condition inspection and morphometrics were normal for the species, the high values observed in these analytes in comparison to other Chondrichthyans could be associated with overfeeding, and further studies involving larger populations of ratfish (including free-ranging individuals) are needed to evaluate if the relatively high values of these analytes are physiologic for the species (2).

Plasma protein electrophoresis values obtained were similar to those previously reported in elasmobranch, in which a fraction migrating in the region equivalent to albumin was not detected in the majority of species studied (13, 28). Fraction 4 (mean 53.2% and 1.55 g/dl) (migrating in the region equivalent to beta-globulins) was the predominant fraction in the Spotted Ratfish, with similar values to those previously described in elasmobranchs such as the Bonnethead Shark (*Sphyrna tiburo*) (mean 47.82% and 1.31 g/dl) and the Atlantic sharpnose Shark (*Rhizoprionodon terranovae*) (mean 44.00% and 1.2 g/dl) (15). Fraction 3 to fraction 4 ratio in the Spotted Ratfish (mean 0.25), which is a ratio used in the clinical evaluation of elasmobranchs as it decreases during inflammatory pathologies, was like that of the clinically healthy nurse shark (mean 0.23) (14, 16). Variations in teleost and elasmobranch electrophoretograms have been associated with changes in diet, fish size, age, season, sex and different inflammatory pathologies (13). Given the important interspecific variations in PPE described in fish, it seems important to determine baseline PPE values in a wide range of Chondrichthyan species, to allow a reliable interpretation of plasma protein electrophoretic variations in the different taxa (13, 16, 24, 29).

## Conclusions

To the authors' knowledge, this is the first study to encompass a detailed description of blood collection, hematology analysis, plasma chemistry and plasma protein electrophoresis values in the *Hydrolagus colliei*. The reported dataset, despite being strongly limited by its small sample size, will contribute to the ongoing discussion on comparative clinical pathology in vertebrates, as well as provide a useful tool for veterinary clinicians working with this species. It is also the first report on induction and recovery times for anesthesia with MS-222 in the Spotted Ratfish, supporting its use in this species when short handling procedures are required.

## Data availability statement

The raw data supporting the conclusions of this article will be made available by the authors, without undue reservation.

## Ethics statement

Ethical approval was not required for the study involving animals in accordance with the local legislation and institutional requirements because blood samples used for this study were obtained from animals sampled in the context of a health management program. In accordance with the European Parliament and Council normative 2010/63/UE (September 22, 2010) on the protection of animals used for scientific purposes, the non-experimental clinical veterinary practice is excluded from the scope of the legislation and therefore approval from the corresponding Ethical Committee was not required.

## Author contributions

PM-E: Conceptualization, Data curation, Formal analysis, Investigation, Methodology, Writing – original draft. HD: Conceptualization, Data curation, Formal analysis, Investigation, Methodology, Validation, Writing – original draft. HB: Methodology, Supervision, Writing – review & editing. VQ: Methodology, Writing – review & editing. NB: Funding acquisition, Supervision, Writing – review & editing. NP: Conceptualization, Data curation, Investigation, Methodology, Supervision, Validation, Writing – review & editing.
