# Supplementary material for: Blood collection under anesthesia, peripheral blood cells, plasma biochemistry, and plasma protein electrophoresis in a living fossil: the Spotted Ratfish (Hydrolagus colliei)
Source: Front Vet Sci. 2024 Jan 11;10:1305968. doi: 10.3389/fvets.2023.1305968 (PMC10808452; doi:10.3389/fvets.2023.1305968)
Supplement: Supplementary file 1 [file Data_Sheet_1.PDF]

*Supplementary Material*

# Blood collection under anesthesia, blood cells, plasma biochemistry and plasma protein electrophoresis in a living fossil: the Spotted Ratfish (Hydrolagus colliei)

**Pablo Morón-Elorza1,2*, Hugo David3, Hugo Batista3, Vanessa Quina3, Nuria Baylina3 and Nuno Pereira3**

*** Correspondence:** Pablo Morón-Elorza: p-moron@hotmail.com

**Supplementary Table 1.** Descriptive data of the three Spotted Ratfish (*Hydrolagus colliei*).

| | Animal 1 | Animal 2 | Animal 3 |
|---|---|---|---|
| Sex | Male | Female | Male |
| Estimated Age (years) | 27 | 27 | 27 |
| Weight (g) | 650 | 1350 | 560 |
| Total length (cm) | 43 | 53 | 54 |
| Snout-vent lenght (cm) | 37 | 46 | 45 |

**Supplementary Table 2.** Time points of anesthesia stages with MS-222 at 50 ppm.[16]

| | Animal 1 | Animal 2 | Animal 3 | Mean | Median |
|---|---|---|---|---|---|
| Excitement phase onset (minutes) | 2.0 | 4.0 | 2.0 | 2.7 | 2.0 |
| Deep sedation onset (minutes) | 4.0 | 6.0 | 5.0 | 5.0 | 5.0 |
| Deep narcosis onset (minutes) | 6.0 | 10.0 | 12 | 9.3 | 10.0 |
| Recovery (minutes) | 13.0 | 20.0 | 28.0 | 20.3 | 20.0 |

**Supplementary Table 3.** Blood cell morphology of the Spotted Ratfish (*Hydrolagus colliei*) from smears stained using Diff Quick and examined at x 100 magnification using immersion oil. For the determination of cell size, a minimum of 30 cells of each type were measured from animal 1, using Image J software and the mean was calculated.

| Cell type | Size/diameter (µm) | Shape | Nucleus | Color (cytoplasm) | Granules |
|---|---|---|---|---|---|
| Erythrocyte | 8.9 x 12.3 | Round, oval | Central, elongated | Pale-gray | None |
| Thrombocyte | 12.0 x 6.0 | Oval, elongated | Central, very elongated | Light-gray | None |
| Lymphocyte | 7.6 x 8.3 | Round | Round, eccentric | Pale-blue | None |
| Monocyte | 12.1 x 11.8 | Round | Kidney- / C-shaped, eccentric | Blue-gray | Basophilic |
| FEG | 13.4 x 12.7 | Round | Shape varies, segmented or not, eccentric | Pale-pink | Few, pink, rod-shaped |
| CEG | 14.2 x 13.8 | Round | Shape varies, segmented or not, eccentric | Colorless | Many, fine, red, round |

FEG, fine eosinophilic granulocyte; CEG, coarsed eosinophilic granulocyte.

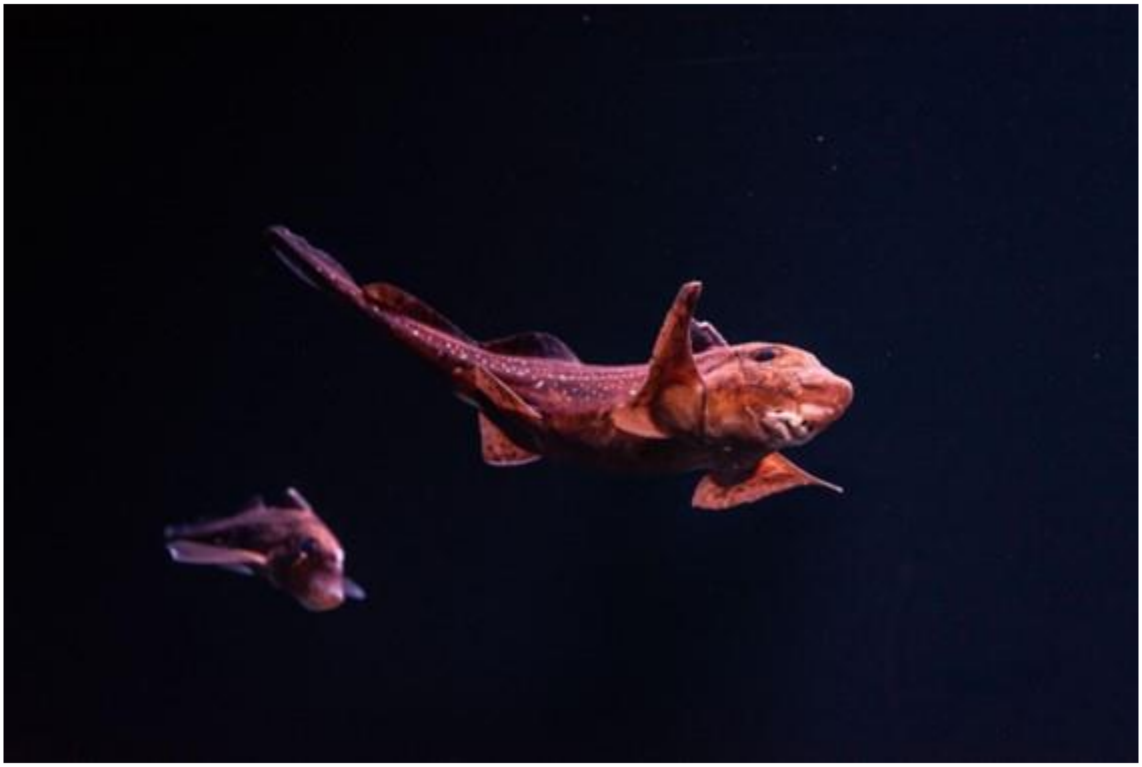

**Supplementary Figure 1.** Two specimens of Spotted Ratfish (*Hydrolagus colliei*), maintained at Oceanário de Lisboa (Portugal. www.oceanario.pt).

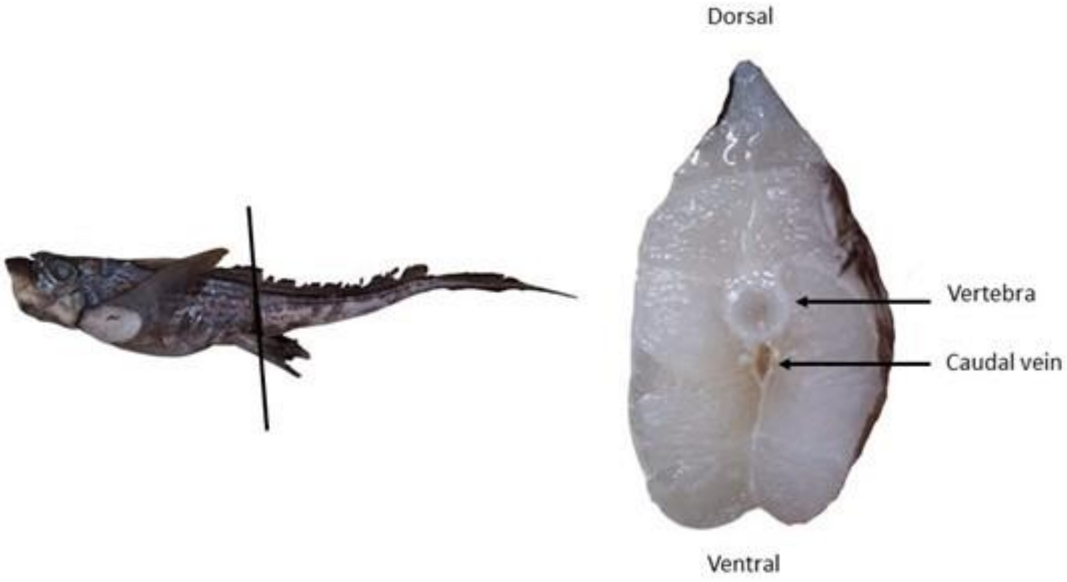

**Supplementary Figure 2.** Photographs of a dead specimen of a *Chimaera monstrosa*. Latero-lateral view (left) and transverse plane (right) showing the anatomical references for blood collection (blood vessels in the caudal hemal canal). Photo kindly shared by David Ruiz García, University of Valencia.

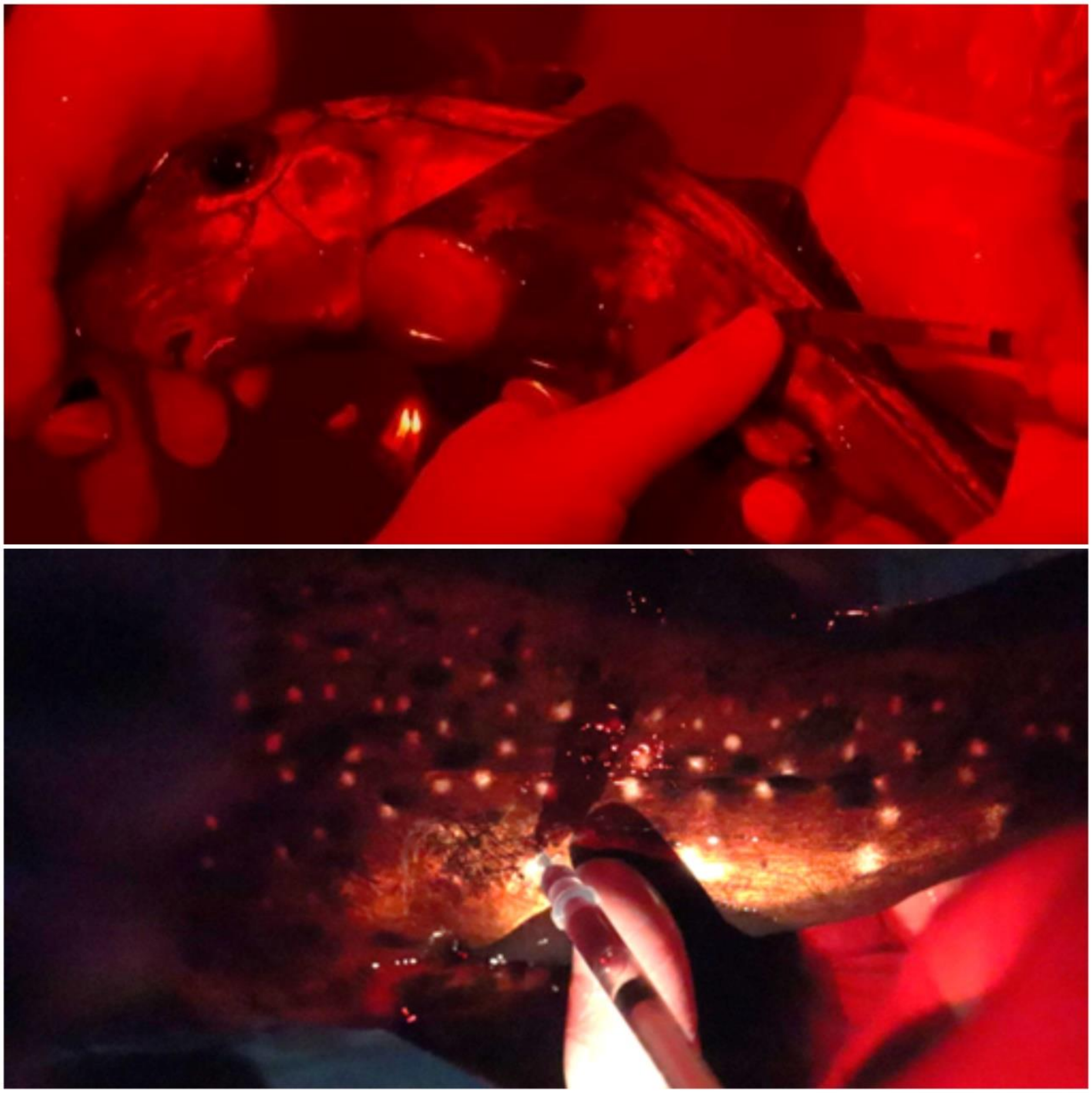

**Supplementary Figure 3.** Peripheral blood collection in a Spotted Ratfish (*Hydrolagus colliei*) using a lateral access of the caudal blood vessel. Please note that to mitigate light related stress, the room lights were off and only a red light was used for blood collection. For a better visualization (inferior image) of the vascular access, a small white light was focused to the needle injection site. Oceanário de Lisboa (Portugal. www.oceanario.pt).

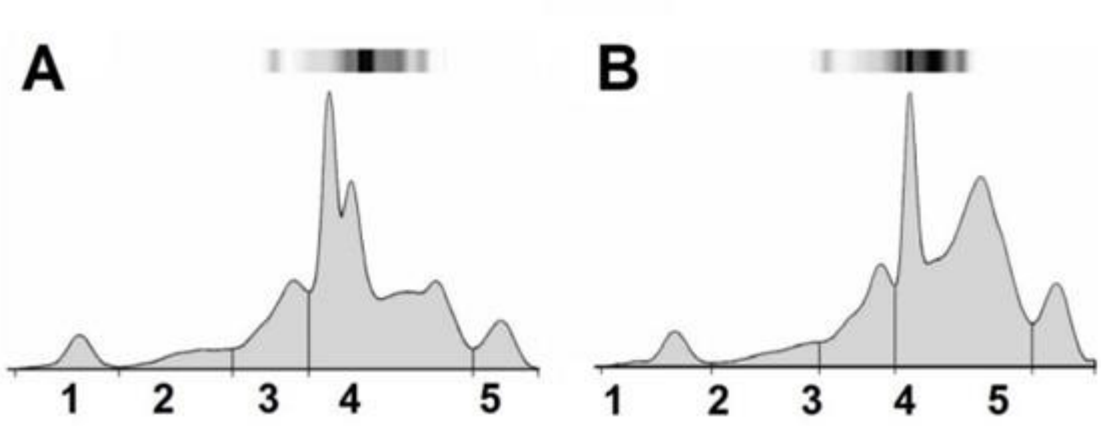


**Supplementary Figure 4.** Representative plasma protein electrophoretogram of two Spotted Ratfish (*Hydrolagus colliei*) (A for animal 1 and B for animal 2). Five consistent fractions are identified, which are migrating in the electrophoretogram regions equivalent to albumin, alpha-1, alpha-2, beta-, and gamma-globulins. A digital representation of the gel-electrophoresis is included to aid visual inspection.
